# Quantitative measurement of alterations in DNA damage repair (DDR) pathways using single cell network profiling (SCNP)

**DOI:** 10.1186/1479-5876-12-184

**Published:** 2014-06-25

**Authors:** David B Rosen, Ling Y Leung, Brent Louie, James A Cordeiro, Andrew Conroy, Iuliana Shapira, Scott Z Fields, Alessandra Cesano, Rachael E Hawtin

**Affiliations:** 1 Research, Nodality Inc., 170 Harbor Way, Suite 200, South San Francisco, CA 94080, USA; 2 Clinical Affairs, Nodality Inc., South San Francisco, CA, USA; 3 Monter Cancer Center, North Shore Long Island Jewish Medical School, 450 Lakeville Road Lake Success, New York, NY 11042, USA

**Keywords:** Genomic instability, DNA damage repair, Cell cycle, Homologous recombination, Non-homologous end joining, BRCA, ATM, PARP, Multiparameter flow cytometry, FACS

## Abstract

**Background:**

Homologous recombination repair (HRR) pathway deficiencies have significant implications for cancer predisposition and treatment strategies. Improved quantitative methods for functionally characterizing these deficiencies are required to accurately identify patients at risk of developing cancer and to identify mechanisms of drug resistance or sensitivity.

**Methods:**

Flow cytometry-based single cell network profiling (SCNP) was used to measure drug-induced activation of DNA damage response (DDR) proteins in cell lines with defined HRR pathway mutations (including *ATM*-/-, *ATM+/-, BRCA1*+/-, *BRCA2*-/-) and in primary acute myeloid leukemia (AML) samples. Both non-homologous end joining (NHEJ) and HRR pathways were examined by measuring changes in intracellular readouts (including p-H2AX, p-ATM, p-DNA-PKcs, p-53BP1, p-RPA2/32, p-BRCA1, p-p53, and p21) in response to exposure to mechanistically distinct genotoxins. The cell cycle S/G2/M phase CyclinA2 marker was used to normalize for proliferation rates.

**Results:**

Etoposide induced proliferation-independent DNA damage and activation of multiple DDR proteins in primary AML cells and *ATM* +/+but not *ATM* -/- cell lines. Treatment with the PARPi AZD2281 +/- temozolomide induced DNA damage in CyclinA2+ cells in both primary AML cells and cell lines and distngiushed cell lines deficient (*BRCA2*-/-) or impaired (*BRCA1*+/-) in HRR activity from *BRCA1*+/+ cell lines based on p-H2AX induction. Application of this assay to primary AML samples identified heterogeneous patterns of repair activity including muted or proficient activation of NHEJ and HRR pathways and predominant activation of NHEJ in a subset of samples.

**Conclusions:**

SCNP identified functional DDR readouts in both NHEJ and HRR pathways, which can be applied to identify cells with *BRCA1+/-* haploinsuffiency and characterize differential DDR pathway functionality in primary clinical samples.

## Background

**Figure 1:**
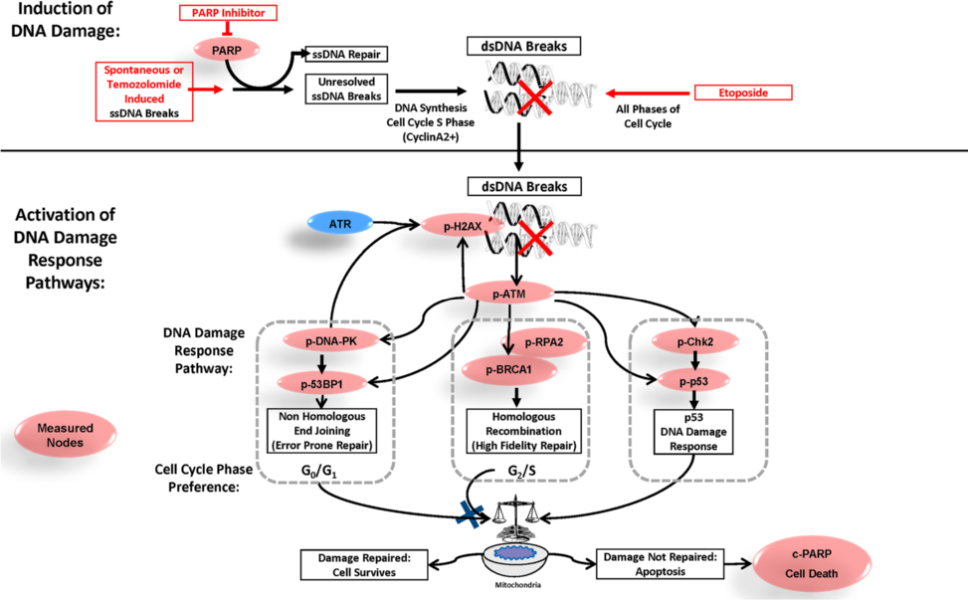
**Overview of measured DDR pathways with focus on repair of DSB.** Experimentally measured parameters are shown in light red.

The DNA damage response (DDR), through the action of sensors, transducers and effectors, orchestrates the appropriate recognition of DNA damage and the repair and resolution of stalled DNA replication [1]. This process is coordinated through complex interplay between cell cycle, apoptosis, and cell survival signaling. The two major mechanisms of DNA double strand break (DSB) repair are homologous recombination repair and non-homologous end joining (HRR and NHEJ, respectively, Figure 1) [1]. HRR predominates in replicating cells and is less error prone, using the identical homologous sister chromatid as a sequence template to repair DSB [2]. HRR is also involved in the repair of interstrand DNA cross-links (ICL) in conjunction with the Fanconi anemia pathway [3]. NHEJ is the predominantly active pathway in resting cells and is the more error-prone mechanism, acting via direct ligation of DNA ends without a homologous template [2,4].

Impaired cellular capacity to repair DNA damage has significant clinical implications, including the development of cancer and the response of cancer cells to cytotoxic therapies [3]. Previous studies have used mutagen sensitivity, host cell reactivation or comet assays to demonstrate associations between functional DNA repair capacity and cancer status or clinical response to chemotherapy or radiation. These data are, however, limited in clinical applicability due to lack of standardization and poor interlaboratory reproducibility [3]. The ability to reproducibly quantify the functional capacity of a cell to repair DNA DSB would enable the identification of individuals at risk of developing cancer and may aid in the selection of appropriate therapeutics for cancer patients.

Single cell network profiling (SCNP) using multiparametric flow cytometry has emerged as versatile tool to quantitatively study the function of multiple specific biological pathways and signaling networks simultaneously using standardized methods and instrumentation [5,6]. By characterizing cellular signaling responses at the single cell level following exposure to extracellular modulators, signaling network integrity and dysfunction can be revealed to identify properties not detectable in resting cells or in the average of the total cell population. Using this functional assay, it is possible to quantitatively measure DNA damage (p-H2AX) in primary clinical samples, such as newly diagnosed adult AML [7]. Herein, SCNP was applied to establish reproducible tools to measure, at the level of the single cell, the functionality of HRR and NHEJ repair pathways with the ultimate goal of identifying functional impairment or deficiency in DNA repair pathways with the potential to inform on drug selection and cancer predisposition.

## Materials and methods

### Experiment overview

This manuscript describes the results of multi-parametric flow cytometry experiments measuring drug-induced DNA damage and activation of multiple DDR readouts in the HRR and NHEJ DSB repair pathways in distinct cell subsets defined based on cell cycle phase to quantify the functional integrity of the cellular DNA repair pathways. Cell lines with functional or defective DNA repair capacities were used to identify reagents capable of detecting activation of DNA damage repair pathways. To confirm that these methods are capable of identifying functional happloinsufficency in a model of *BRCA1* inherited germline mutation, cell lines of known HRR status (*BRCA1*+/- and *BRCA1*+/+) were assessed. Lastly, primary AML samples were used to demonstrate the ability to quantify activation of these pathways in primary cancer cells.

### Flow sample and specimen description

#### Cell lines

Individual cell lines harboring mutations in *ATM, BRCA2* or *BRCA1* were used in the study. Following the suppliers’ instructions, cell lines obtained from ATCC (Manassas, VA) were maintained in RPMI-1640 supplemented with 10% FCS, and cell lines obtained from Coriell Cell Repositories (Camden, New Jersey) were maintained in complete RPMI-1640 supplemented with 15% FCS.

Cell line panel 1 was used to establish tools for measuring ATM-dependent DNA damage responses; *ATM* wild type cell lines [U937 and RS4;11 (ATCC), GM00536 and GM09703 (Coriell)], were compared with an *ATM* +/- cell line [GM03323 (Coriell)] and two *ATM*-/- cell lines [GM01526 and GM03189 (Coriell)] for measurement of etoposide-induced DDR readouts (details in SCNP assay section below).

Cell line panel 2 was used to detect differences between *BRCA2*-/-, *BRCA1*+/-, and *BRCA1*+/+ samples in response to PARPi treatment (details in SCNP assay section below). To model *BRCA2-/-* status, an Epstein Barr Virus (EBV)-transformed B lymphocblast cell line [GM13023 (Coriell)] from a Fanconi’s Anemia patient with homozygous *BRCA2/FANCD1* mutation was used. To model heterozygous *BRCA1* mutation (*BRCA1* +/-), 5 EBV-transformed B lymphoblast cell lines [HCC1937BL (ATCC), GM14091, GM13705, GM13709 and GM14090 (Coriell)] from patients with *BRCA1*-mutated familial breast cancer were used. Similarly, to model wild type *BRCA1* (*BRCA1* +/+) status, 5 EBV-transformed B lymphoblast cell lines [(HCC1954BL (ATCC), GM00536, GM005423, GM17230 and GM17217 (Coriell)] from breast cancer patients whose tumors are negative for *BRCA1* mutations or from healthy donors were evaluated.

#### Patient samples

AML samples consisted of either peripheral blood mononuclear cell (PBMC) or bone marrow mononuclear cell (BMMC) specimens obtained from pediatric or adult patients with AML. Mononuclear cells were purified by ficoll centrifugation then cryopreserved in 90% FBS, 10% DMSO. In accordance with the Declaration of Helsinki, all patients consented to the collection of biospecimens for biology studies.

### Sample processing and instrument details

#### SCNP assay

SCNP assays were performed as described previously [8]. Aliquots of cryopreserved cells were thawed at 37°C, washed, resuspended in RPMI-1640 medium supplemented with 60% fetal bovine serum (FBS), and live mononuclear cells isolated via ficoll density gradient. After a second washing step with RPMI-1640 60% FBS, cells were washed in RPMI-1640 10% FBS, counted, filtered, re-suspended in RPMI-1640 10% FBS, then aliquoted (100,000 cells/condition for primary AML cells or 50,000 cells/condition for cell lines) and rested for 30 minutes at 37°C before addition of therapeutic agents (each tested at a clinically relevant dose ranging between Cmax and trough level as reported in pharmacokinetic studies [9-11]). For all conditions, following incubation with drugs, cells were stained with amine aqua viability dye (Life Technologies, Carlsbad, CA) to distinguish non-viable cells, fixed with 1.6% paraformaldehyde for 10 minutes at 37°C, pelleted, permeabilized with 100% ice-cold methanol, and stored at -80°C. For antibody staining, cells were washed with FACS buffer (PBS, 0.5% BSA, 0.05% NaN_3_), pelleted, and stained with unlabeled antibody cocktails followed by fluorochrome conjugated goat anti mouse or goat anti rabbit secondary antibodies (Life Technologies and Jackson Immunoresearch, West Grove, PA), then blocked with normal rabbit serum and normal mouse serum (Life Technologies) and stained with cockails of fluorochrome-conjugated antibodies. Cocktails included antibodies against cell surface markers for cell gating of AML cells [e.g. CD45, CD11b (Beckman Coulter, Brea, CA), CD34 and CD33 (BD Biosciences, San Jose, CA)] and up to 3 antibodies against intracellular signaling molecules (detailed below) for 6- 8-color flow cytometry assays.

Data was acquired on an LSR II flow cytometer using the FACS DIVA software (BD Biosciences). All flow cytometry data were analyzed with FlowJo (TreeStar Software, Ashland, OR) or WinList (Verity House Software, Topsham, ME). Daily QC of the LSRII cytometers was performed as previously described [12]. Dead cells and debris were excluded by forward and side scatter properties combined with amine aqua viability dye exclusion. For AML samples, “all” non-apoptotic leukemic cells were identified based on expression of CD45 and side-scatter properties and lack of the apoptosis marker cleaved PARP (cPARP, BD Biosciences) as previously described [8,13], while CyclinA2 (Beckman Coulter) staining discriminated CyclinA2- and CyclinA2+ subsets. Similarly, normal lymphocytes within AML samples were identified by low side scatter and high CD45 expression as previously described [8,13]. For cell lines, forward scatter, side scatter, amine aqua, and cleaved PARP similarly identified live non-apoptotic (healthy) cells and CyclinA2 staining discriminated CyclinA2- and CyclinA2+ subsets. Specific drug treatments and readouts examined were as follows:

a) For experiments measuring multiple DDR readouts after etoposide treatment, cell lines (Cell line panel 1) or primary AML samples were treated with 30 μg/mL etoposide (Sigma, St. Louis, MO) for 2 h or 6 h and assayed for p-BRCA1 (S1423) (Novus, Littleton, CO), pDNA-PKcs (T2609) (Biolegend, San Diego, CA), p-53BP1 (S1778), p-ATM (S1981), p-p53 (S15), p-Chk2 (T68), and p-H2AX (S139) (Cell Signaling Technologies, Danvers, MA).

b) For experiments showing magnitude and reproducibility of multiple AZD2281+/- temozolomide-induced DDR readouts and the ability of these readouts to stratify for HRR status, *BRCA1* +/+ , *BRCA1* +/- , and *BRCA2* -/- cell lines (Cell line panel 2) were treated with 6 μg/mL AZD2281 (Selleck, Houston, TX) +/- 2 μg/mL temozolomide (Sigma) for 48-72 h and assayed for p-H2AX (S139), p-RPA2/32 (T21) (Abcam, Cambridge, MA), p-DNA-PKcs (T2609), p21, p-ATM (S1981) and p-BRCA1 (S1423) in CyclinA2- and CylcinA2+ cells. In these experiments a 2 μg/mL dose of temozolomide was used due to excessive apoptosis observed with the combination of AZD2281 with higher doses of temozolomide (10 μg/mL) at later timepoints (data not shown).

c) For experiments measuring the cell cycle selectivity of individual genotoxic agents, cell lines in culture or AML samples pre-treated for 48 h with a panel of myeloid growth factors to induce proliferation in all AML subtypes [20 ng/mL of IL-3 (BD Biosciences), TPO, SCF (R&D Systems, Minneapolis, MN) and FLT3L (ebiosciences, San Diego, CA)] were challenged for 6 h with etoposide (30 μg/mL), PARP inhibitor AZD2281 (6 μg/mL), temozolomide (10 μg/mL), or the combination of AZD2281 + temozolomide (6 μg/mL + 10 μg/mL) and assessed for induced p-H2AX in distinct subsets of cells including CyclinA2+ cells, CyclinA2- cells, or “All” live, cPARP- cells regardless of CyclinA2 status.

### Data analysis details

#### Metrics

**Figure 2:**
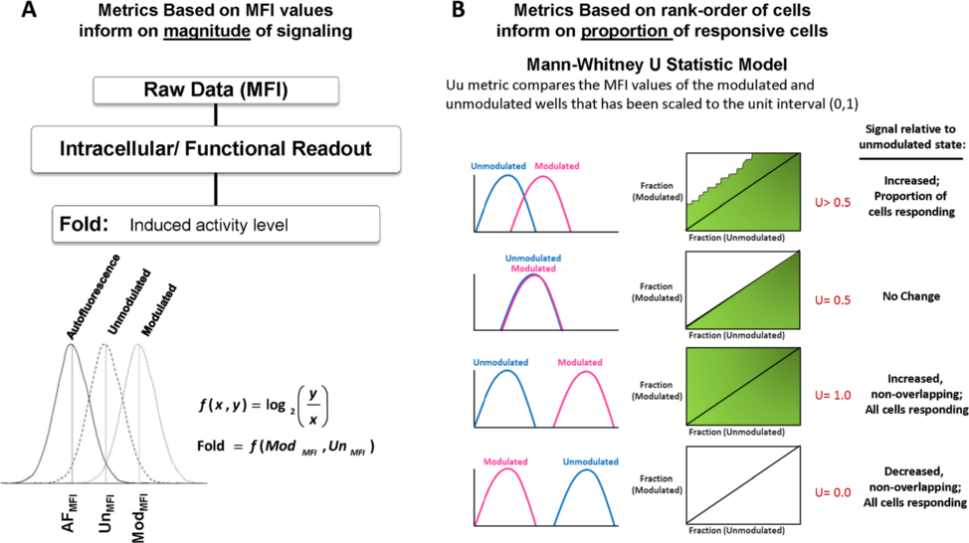
**Illustration of metrics used to quantify DDR responses.** Two classes of metrics are calculated to measure distinct biological responses to modulation. The magnitude of the cellular responses is measured using **(A)** the Log_2_Fold metric while the fraction of cells responding is measured by the **(B)** Uu metric. **(B)** Examples of signaling responses for different Uu values are shown. Top to Bottom: Induced signaling will yield Uu values > 0.5; Lack of intracellular cellular response to modulation will yield Uu values ~ 0.5; Robust increases in signaling result in large magnitude shifts and a maximum Uu value of 1.0; Robust decreases in signaling result in large magnitude shifts with a minimum Uu value of 0.0.

Metrics, illustrated in Figure 2, for quantifying DDR (Log_2_Fold and Uu) were applied to gated cell populations containing at least 200 events and have been described previously [8,14]. Briefly, the Log_2_Fold metric measures the magnitude of the responsiveness of a cell population to modulation where a value of zero indicates lack of induced signaling while a positive value indicates an increase in the signaling response of a population. The “Uu” metric measures the proportion of responsive cells by comparing the overlap and rank-order of the modulated and unmodulated populations on a cell-by-cell basis and ranges from zero to one where 0.5 indicates no change, values >0.5 indicate cells have increased in signal, and values < 0.5 indicate that cells have decreased in signal vs. an unmodulated population. The U metric is useful for comparing data on a normalized scale of zero to one and was used here to compare DDR readouts in AML samples and to assess the cell cycle selectivity of various drugs by comparing the proportion of responding cells within CyclinA2- or CyclinA2+ populations for each agent. Population frequency metrics for the percentage of healthy non-apoptotic (Aqua-, cPARP-) cell line or leukemic cells were calculated. For both cell lines and AML samples, percentages of CyclinA2- or CyclinA2+ cells of the parent population were calculated. For both cell lines and AML samples DDR metrics were calculated for “all” healthy non-apoptotic cells as well as CyclinA2- and CyclinA2+ subsets where possible.

#### Statistics

Individual DDR readouts were correlated against each other using Pearson’s linear correlation.

Data on drug-induced cytotoxicity and DNA damage were correlated with proliferation levels using Pearson’s linear correlation. Comparison of CyclinA2+ vs CyclinA2- cells for magnitude and CV of signaling was performed with using a paired student’s t-test. Student’s t-test was performed to compare *BRCA1*+/- vs. *BRCA1*+/+ cell line responses.

## Results

### Development of SCNP assays to quanitfy induction of DNA damage and ATM mediated activation of DNA damage repair pathways

**Figure 3:**
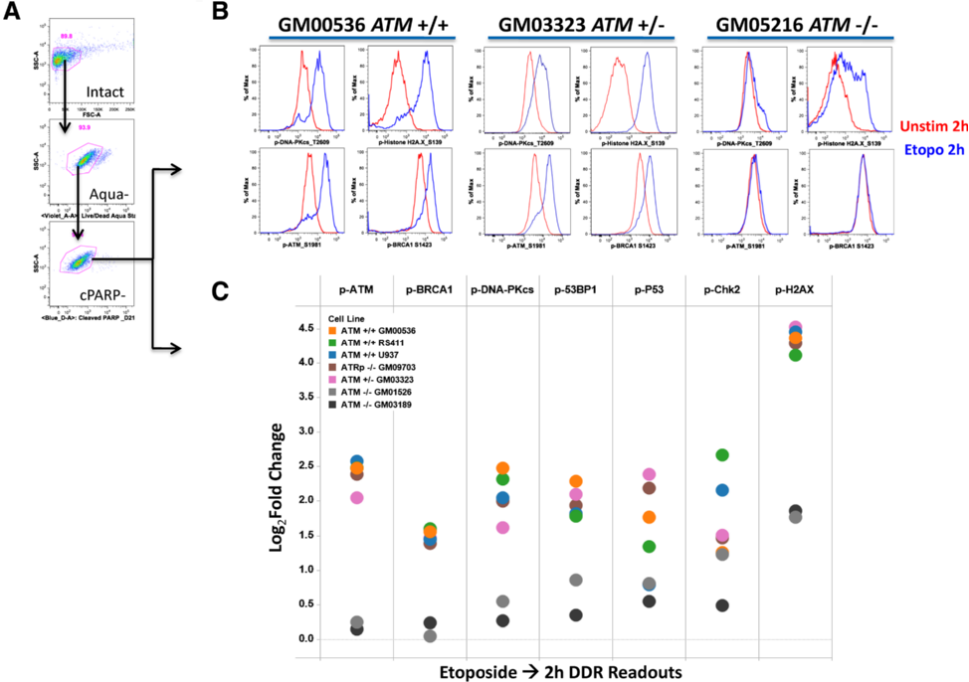
**Analysis of etoposide inducible SCNP DDR readouts in cell lines with characterized DDR pathway deficiencies. (A)** Flow plots illustrate the gating scheme to identify live (Aqua-) non-apoptotic, healthy (cPARP-) cells for analysis of DDR responses. **(B)** Flow plot examples of p-DNA-PKcs, p-H2AX, p-ATM and p-BRCA1 induction in *ATM+/+, ATM+/-* and *ATM*-/- cell lines (Red: 2 h unstimulated, Blue: 2 h etoposide treated, (30 μg/mL)). **(C)** Shown are Log_2_Fold change in levels of intracellular readouts following 2 hr etoposide treatment. DDR readouts: p-ATM, p-BRCA1, p-DNA-PKcs, p-53BP1, p-p53, p-Chk2, and p-H2AX. Cell lines (Cell line panel 1, Materials and methods) are coded by color. *ATM*-/- cell lines (grey and back dots) consistently show lower induced values for all modulated DDR readouts measured. In contrast, *ATM* +/- cells (magenta dots) shows slightly lower DDR readouts vs. *ATM* +/+ cells for some (p-ATM, p-DNA-PKcs) but not all reaoduts in these conditions.

To quantitatively assess induction of DNA damage and activation of DDR pathways at the single cell level, reagents and experimental conditions were first optimized to detect the induction of activation motifs within the two major double strand break repair pathways: NHEJ and HRR. Using the topoisomerase II inhibitor etoposide to induce DDR pathway activation and DNA damage, reagents recognizing the induced phosphorylation sites on multiple proteins within the NHEJ and HRR pathways were examined (Figure 1). In a panel of cell lines containing defined mutations including *ATM-/-, ATM+/-,* and *ATM+/+* genotypes (Cell line panel 1), etoposide induced phosphorylation and activation of all proteins tested. DDR response data for each *ATM* genotype and an illustration of the gating scheme used are shown in Figure 3. Consistent with the functional role of ATM in mediating signaling through both HRR and NHEJ pathways, etopside treatment induced lower activation of all readouts in the *ATM*-/- cell lines compared to *ATM*+/- and *ATM+/+ *cell lines and showed slightly lower activation of the *ATM*+/- cell line vs. *ATM*+/+cell lines for two readouts (p-ATM and p-DNA-PKcs). Of note, no significant differences were seen comparing the percentages of live, healthy cells or CyclinA2+ cells between *ATM-/- , ATM+/-* and *ATM+/+ *genotypes (data not shown), suggesting that DDR pathway measurements may be more informative than general measures of cell death/cell cycle status. This supports the utility of the evaluated DDR readouts (nodes) to quantify ATM mediated activation of DNA damage response pathways.

### Quantification of PARPi +/- TMZ induced p-H2AX levels in CyclinA2+ cells robustly identifies alterations in HRR pathway activity

The ability of SCNP to measure DDR responses in cellular subpopulations was next exploited as a method to quantify the activity of cell cycle specific DDR pathways such as HRR. To identify robust DDR readouts which could be leveraged to recognize cells with functionally defective or impaired HRR pathway activity, the cellular response to PARPi AZD2281 was examined using an expanded panel of DDR readouts and cell lines with known mutations in HRR machinery, including *BRCA2*-/-, *BRCA1+/-,* and *BRCA1+/+ *lines (Cell line panel 2). Cells were treated with PARPi (AZD) +/- temozolomide (TMZ) for 48-72 h and analyzed for the induction of 6 DDR proteins [p-H2AX, p-RPA2, p-DNAPKcs, p-ATM, p-BRCA1 and p21 (total protein)] in all cells (regardless of CyclinA2 expression), in CyclinA2- and in CyclinA2+ cell subsets within the same cell line. This choice of conditions was based on previous experiments in cell lines demonstrating that PARPi AZD2281 +/- temozolomide selectively induced DSBs (p-H2AX) in CyclinA2+ cells while etoposide induces DSBs (p-H2AX) in both CyclinA2- and CyclinA2+ cells (data not shown).

**Figure 4:**
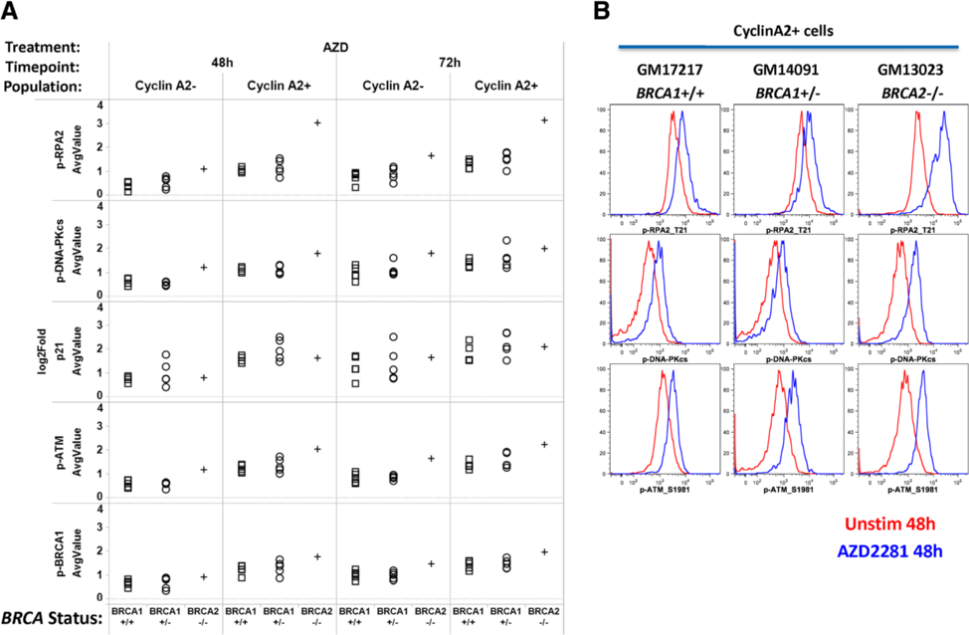
**Analysis of PARPi-induced DDR in cell lines shows higher induction of multiple readouts in *BRCA2-/- *cells compared to *BRCA1+/- *and *BRCA1+/+*cells. (A)** 48 or 72 hr (columns) PARPi AZD2281 (AZD, 6 μg/mL)-induced Log_2_Fold values for DDR readouts p-RPA2, p-DNA-PKcs, p21, p-ATM, and p-BRCA1 (rows) are shown for CyclinA2- or CyclinA2+ subsets (columns) of tested cell lines. Values shown are mean averages from 2 independent experiments. Cell lines are coded by HRR status (*BRCA1*+/+, squares; *BRCA1*+/-, circles; *BRCA2-/-*, crosses). **(B)** Flow plot examples of p-RPA2, p-DNA-PKcs, p-ATM induction in *BRCA1*+/+, *BRCA1*+/-, and *BRCA2*-/- cell lines at 48 h (Red: Unstimulated, Blue: AZD2281).

PARPi-induced DDR responses from all cells, CyclinA2-, and CyclinA2+ subsets were examined in the HRR mutant and proficient cell lines in the context of their HRR status. Elevated levels of PARPi-induced p-ATM, p-BRCA1, p-RPA2, and p-DNA-PKcs were detected in the *BRCA2-/-* cell line in CyclinA2+ cells at both 48 h and 72 h (Figure 4). *BRCA1+/-* vs *BRCA1+/+* cell lines were not distinguished by readouts for PARPi +/- TMZ-induced p21, p-ATM, p-BRCA1, p-RPA2, and p-DNA-PKcs in any population analyzed (Table 1). Although some *BRCA1+/-* samples demonstrated a trend of higher PARPi-induced p21 levels vs. *BRCA1+/+* samples (Figure 4), the difference was not significant (Table 1).

**Table 1:** **T-Test P-values comparing DDR readouts between *BRCA1*** **+/- vs. *BRCA1*** **+/+ cell lines**

**Treatment**	**AZD**			**AZD**			**AZD + TMZ**			**AZD + TMZ**		
**Timepoint:**	**48 h**			**72 h**			**48 h**			**72 h**		
**Population:**	**All cells**	**Cyclin A2-**	**Cyclin A2+**	**All cells**	**Cyclin A2-**	**Cyclin A2+**	**All cells**	**Cyclin A2-**	**Cyclin A2+**	**All cells**	**Cyclin A2-**	**Cyclin A2+**
Stain:												
p-H2AX	0.536	0.119	**0.012**	0.558	0.151	**0.039**	0.673	0.172	**0.014**	0.509	0.272	**0.011**
p-RPA2	0.545	0.372	0.545	0.599	0.442	0.229	0.875	0.445	0.916	0.900	0.767	0.859
p-DNA-PKcs	0.398	0.419	0.957	0.597	0.469	0.336	0.776	0.952	0.894	0.758	0.562	0.297
p21	0.315	0.413	0.132	0.645	0.737	0.203	0.693	0.708	0.364	0.778	0.706	0.452
p-ATM	0.636	0.848	0.390	0.940	0.693	0.228	0.746	0.967	0.736	0.813	0.920	0.694
p-BRCA1	0.754	0.954	0.608	0.505	0.872	0.721	0.858	0.839	0.756	0.684	0.970	0.860

Statistically significant p-values (p < .05) for stratifying BRCA1+/- vs. BRCA1+/+cell lines are shown in bold.

**Figure 5:**
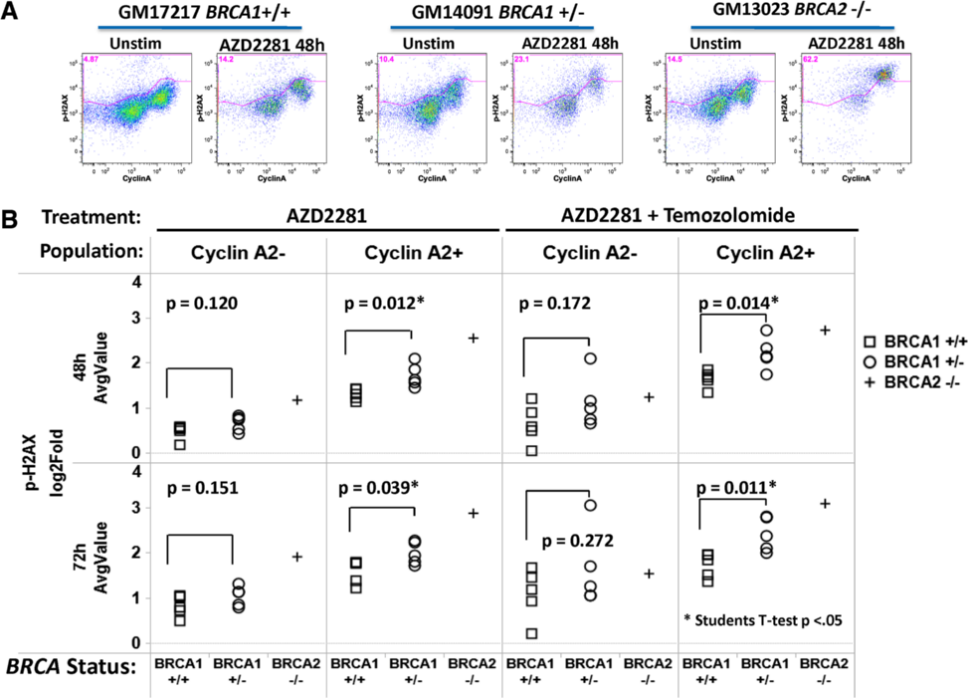
**Analysis of PARPi-induced p-H2AX in CyclinA2- and CyclinA2+ subsets in DDR wild type or mutant cell lines distinguishes distinct levels of HRR deficiency including haploinsufficiency. (A)** Flow plot examples of p-H2AX vs. CyclinA2 in untreated or 48 h PARPi AZD2281 (6 μg/mL) treated *BRCA1*+/+, *BRCA1*+/- and *BRCA2*-/- cell lines. **(B)** PARPi AZD2281 +/- temozolomide (2 μg/mL)-induced p-H2AX in CyclinA2- or CyclinA2+ subsets (columns) after treatment for 48 or 72 hr (rows) for cell lines of known HRR status (*BRCA1*+/+, squares; *BRCA1*+/-, circles; *BRCA2-/-*, crosses; Cell line panel 2, Materials and methods). Mean average Log_2_Fold values from two independent experiments are shown. T-test p-valuescomparing *BRCA1*+/+and *BRCA1*+/- cell lines are shown for each condition with significant (p < .05) indicated by an asterisk (*). Analysis of AZD2281 +/- temozolomide treatment in CyclinA2+ cells demonstrates highest p-H2AX induction in the *BRCA2*-/- cell line, medium induction in *BRCA1+/-* cell lines and lowest induction in *BRCA1*+/+ cell lines.

The most sensitive readout for HRR pathway function was p-H2AX. Analysis of CyclinA2+ cells consistently demonstrated highest induced p-H2AX levels in the homozygous *BRCA2*-/- cell line, intermediate p-H2AX levels in heterozygous *BRCA1*+/- cell lines, and lowest p-H2AX levels in *BRCA1*+/+ cell lines (Figure 5). Importantly, gating on CyclinA2+ cells distinguished *BRCA1+/-* from *BRCA1+/+* lines; p-H2AX levels were significantly (p < .05) higher in *BRCA1*+/- lines compared to *BRCA1*+/+ lines in CyclinA2+ cells but not in CyclinA2- cells or in the parent population at all timepoints and treatment conditions tested (Figure 5, Table 1). These data suggest that analysis of DDR readouts specifically in CyclinA2+ cells enables more accurate quantification of HRR proficiency, possibly by reducing (and normalizing for) the confounding effects of different proliferation rates across samples.

To further examine CyclinA2 gating as a method to control for proliferation rate, the above PARPi-induced DDR data from *BRCA2*-/-, *BRCA1+/-,* and *BRCA1 +/+ *cell lines were evaluated for the percentage of CyclinA2+ cells as a measure of proliferation for each cell line and used to compute correlations between proliferation and PARPi +/- temozolomide-induced DDR readouts measured in three populations: a) CyclinA2+ cells only, b) CyclinA2- cells only or b) all healthy cells. While DDR readouts measured in all live cells or CyclinA2- cells demonstrated higher correlations with proliferation (average R^2^ values: 0.403, 0.505, respectively), DDR readouts from CyclinA2+ subsets were less correlated with proliferation (average R^2^: 0.194). These data confirm that analysis of DDR readouts specifically in CyclinA2+ cells helps normalize for cell proliferation rate, enabling more accurate quantification of HRR proficiency. In addition, examination of the percentages of live, healthy cells with or without PARPi treatment demonstrated no significant differences in apoptosis between *BRCA1*+/+ and *BRCA1*+/- samples (data not shown), which is consistent with published literature [15,16], and suggests that DDR pathway measurements may be more sensitive than apoptosis measurements in identifying altered DNA repair phenotypes.

To further understand the technical components of quantifying PARPi-induced DDR readouts in CyclinA2- or CyclinA2+ cells, the magnitude and reproducibility of DDR readouts were directly compared between CyclinA2- and CyclinA2+ subsets.

**Figure 6:**
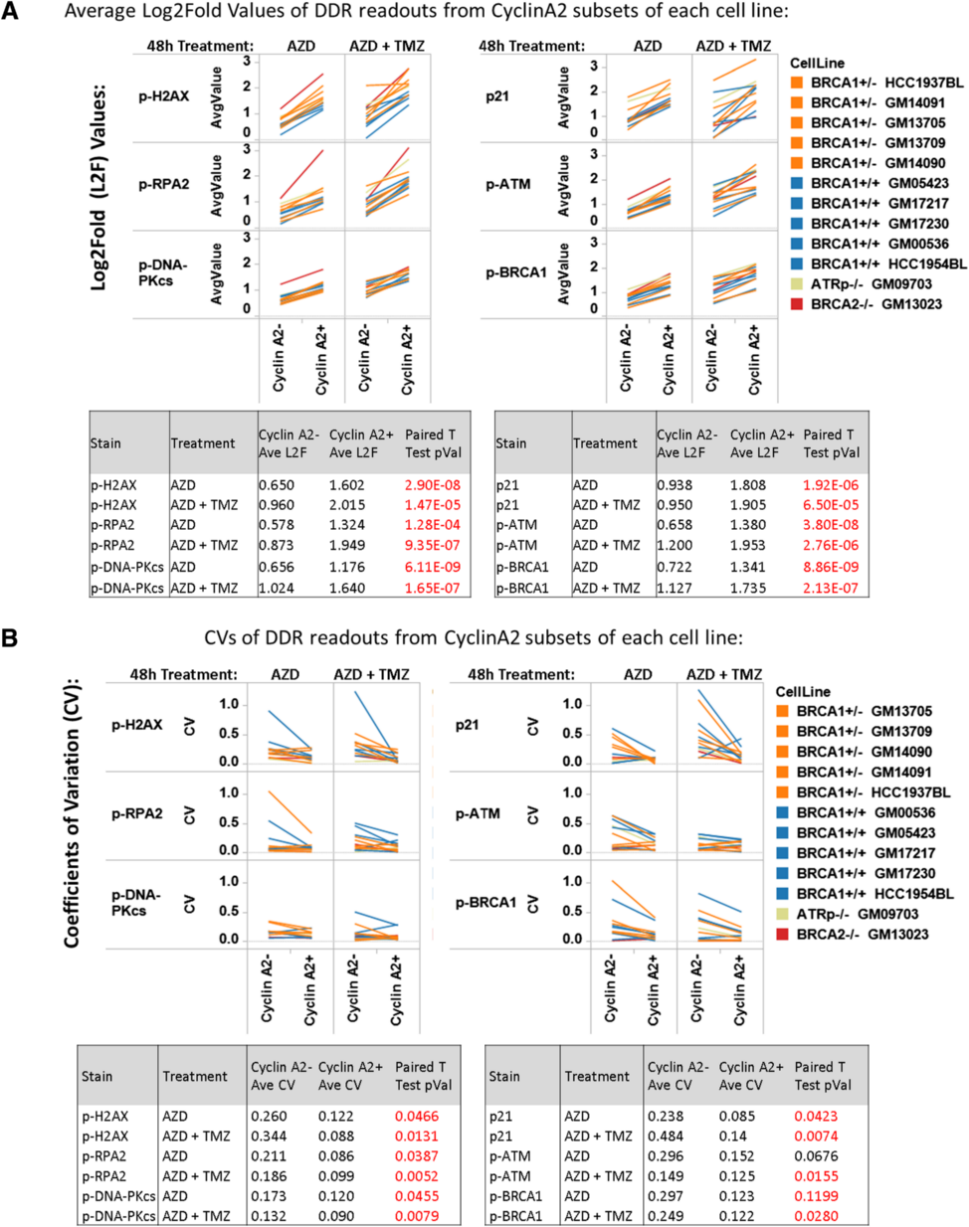
**Analysis of magnitude and reproducibility of PARPi-induced DDR readouts in CyclinA2+ vs CyclinA2- cells. (A)**. DDR wild type or mutant EBV cell lines (*ATRp*-/-, *BRCA2-/-, BRCA1+/-, BRCA1+/+* genotypes*,* Cell Line panel 2, Materials and methods) were treated with PARP inhibitor AZD2281 (AZD, 6 μg/mL) or the combination of AZD2281 + temozolomide (AZD + TMZ, 6 μg/mL + 2 μg/mL, respectively) (columns) for 48 hr and assayed for activation of DDR readouts. Shown are Log_2_Fold values measuring the magnitude of modulation of p-H2AX, p-RPA2, p-DNA-PKcs (left) or p21, p-ATM, p-BRCA1 (right) in both CyclinA2- and CyclinA2+ subsets (top). Tabulated below are the average Log_2_Fold (L2F) values for each readout and treatment condition in CyclinA2- and CyclinA2+ subsets, with the paired t-test p-value comparing CyclinA2- and CyclinA2+ L2F values. Data shown are the average of two experiments **(B)**. Shown are Coefficient of Variation (CV) values measuring the reproducibility between the two experiments shown in **(A)**. CVs were computed for CyclinA2- and CyclinA2+ populations for each readout and treatment condition. Tabulated below are the average CVs for each readout and treatment condition in CyclinA2- and CyclinA2+ subsets and the paired t-test p-value comparing CyclinA2- and CyclinA2+ CVs.

As shown in Figure 6A, the magnitudes of PARPi (AZD) +/- TMZ-induced DDR responses were statistically higher for all 6 DDR readouts in CyclinA2+ vs. CyclinA2- cells (paired t-test p-values directly comparing CyclinA2- and CyclinA2+ data ranged from p = 1.28 × 10^-4^ to p = 8.86 × 10^-9^). Within the CyclinA2+ population, p-H2AX and p21 showed highest induction of signal among the DDR readouts. Higher levels of DDR readouts were observed in conditions with temozolomide in both CyclinA2- and CyclinA2+ cells.

To assess the reproducibility of these data, the coefficient of variation (CV) between two independent experiments was computed. As shown in Figure 6B, better reproducibility was observed for all 6 DDR readouts in CyclinA2+ (average CV: 0.113) vs. CyclinA2- cells (average CV: 0.252) in all treatment conditions (+/- TMZ), with significant differences (p < .05 paired t-test) in CVs observed between CyclinA2+ and CyclinA2- populations for 11/12 conditions tested. Of note, these CVs are similar to values observed with comparable quantitiative flow cytometery bio-assays such as induction and detection of intracellular cytokines (average CV from manual gating: 0.28) [17].

### Assessment of DNA damage and activation of DNA damage repair pathways in primary AML samples

**Figure 7:**
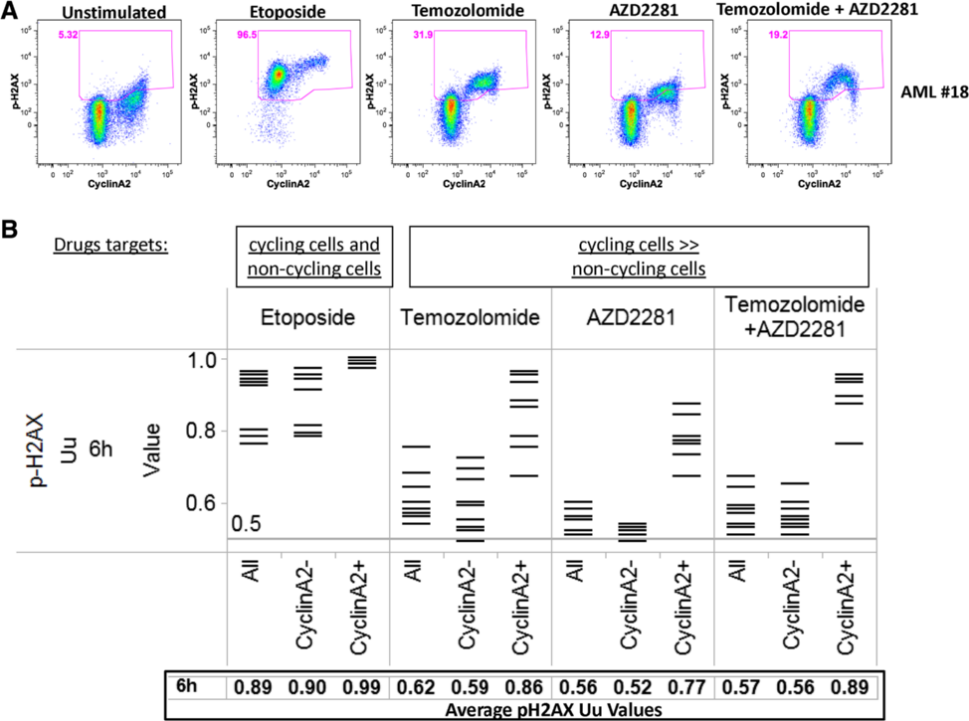
**Analysis of DDR in CyclinA2+ and CyclinA2- cells demonstrates cell-cycle specific effects of individual genotoxins in AML samples.** Primary AML (PBMC and BMMC) samples were treated with etoposide (30 μg/mL), PARP inhibitor AZD2281 (6 μg/mL), temozolomide (10 μg/mL), or the combination of AZD2281 + temozolomide (6 μg/mL + 10 μg/mL) for 6 hr. **(A)** Example flow plots of CyclinA2 vs. p-H2AX for all leukemic cells of a representative AML sample. **(B)** Shown is the proportion of cells (Uu metric) with induced p-H2AX in each sample; this metric allows comparisons of induced DDR from different drugs on a normalized scale. Modulated signaling through p-H2AX was measured in the bulk leukemic cell population (All cells) or in CyclinA2- or CyclinA2+ subsets. Tabulated below are the average values of all AML samples in each population.

**Figure 8:**
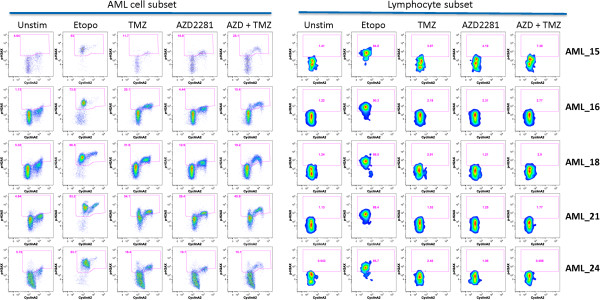
**Genotoxin effects on CyclinA2 subets of leukemic cells or lymphocytes from myeloid growth factor stimulated AML samples.** Shown are flow plot examples of CyclinA2 vs. p-H2AX for leukemic AML cells (left) or normal lympohcytes (right) gated (see Materials and methods) from 5 AML samples treated with etoposide (30 μg/mL), PARP inhibitor AZD2281 (6 μg/mL), temozolomide (10 μg/mL), or the combination of AZD2281 + temozolomide (6 μg/mL + 10 μg/mL) for 6 hr. While leukemic CyclinA2+ cells show induced p-H2AX from all agents tested, leukemic and lymphocytic CyclinA2- cells only demonstrate induced p-H2AX after etoposide treatment.

**Figure 9:**
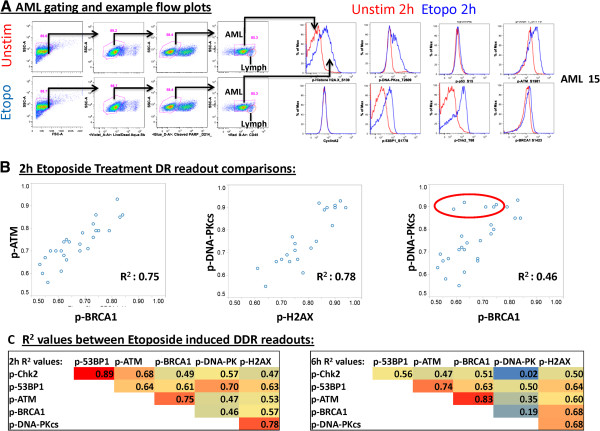
**Analysis of correlations between etoposide-induced DDR nodes in AML samples highlights unique patient biology.** Primary AML (PBMC and BMMC) samples were treated with 30 μg/ml etoposide for 2 hr or 6 hr and the modulation of a panel of DDR readouts was measured (p-53BP1, p-ATM, p-BRCA1, p-DNA-PKcs, p-H2AX). **(A)** Flow plots illustrating the gating scheme for leukemic cells and lymphocytes from AML samples (left) and etoposide induced DDR responses in leukemic cells for p-H2AX, p-DNA-PKcs, p-p53, p-ATM, p-53BP1, p-Chk2 and p-BRCA1 (right). **(B)** Individual modulated DDR readouts were then compared to each other with bivariate plots, with R^2^ values shown. While higher correlations were observed between the DSB response (p-ATM) and HRR pathways (p-BRCA1) (left scatter plot), and between measures of DNA damage (p-H2AX) and NHEJ repair (p-DNA-PKcs) (middle scatter plot), lower correlations were observed across pathways comparing HRR (p-BRCA1) and NHEJ (p-DNA-PKcs) readouts (right scatter plot). Samples enclosed by a red circle demonstrate relatively higher activation of NHEJ pathway components (p-DNA-PKcs) as compared to HRR pathway components (p-BRCA1). **(C)** Correlation R^2^ values between etoposide induced DDR readouts at 2 h and 6 h.

To test the ability of SCNP to measure cell-cycle specific DDR responses to specific genotoxic agents in primary samples, peripheral blood or bone marrow samples from patients with AML were stimulated to proliferate by 48 h incubation with a panel of myeloid growth factors then evaluated for p-H2AX induction after 6 h in vitro treatment with etoposide, PARPi, TMZ, or the combination of PARPi + TMZ. As expected, growth factor treatment stimulated AML cells to enter the cell cycle as measured by the frequency of CyclinA2+ cells (Figures 7 and 8). Of note, the frequency of CyclinA2+ cells after growth factor treatment was similar among AML samples (median: 25.0%, standard deviation: 8.0%). Similar to results seen with cell lines (data not shown), treatment of primary AML samples with etoposide resulted in decreased numbers of CyclinA2+ cells available for analysis and induced DNA damage (p-H2AX) in both CyclinA2- and CyclinA2+ AML cell subsets (Figure 7). In contrast, treatment of primary AML samples with TMZ, PARPi, or the combination of PARPi + TMZ-induced proliferation-dependent DNA damage (p-H2AX) selectively in CyclinA2+ AML cells, again consistent with the mechanism of action of these agents. Example flow plots for four additional AML samples are shown in Figure 8 and an illustration of of gating for leukemic or lymphocyte cell subsets is shown in Figure 9. Interestingly, analysis of the lymphocyte cell responses from AML samples demonstrated that; 1) as expected lymphocytes did not respond to the myeloid growth factors and lacked CyclinA2 expression and; 2) in agreement with the results from analysis of gated AML cells, etoposide induced a robust p-H2AX response in CyclinA2- lymphocytes while treatment with TMZ, PARPi or PARPi + TMZ had minimal effects on CyclinA2- lymphocytes (Figure 8).

A larger panel of markers was then examined in the context of primary AML, measuring both NHEJ and HRR pathway activity in response to in vitro etoposide treatment for 2 h or 6 h. As etoposide and other genotoxic agents are currently part of standard AML chemotherapy regimens [18,19], examination of etoposide-induced DDR readouts in AML samples may be clinically relevant. As etoposide treatment was previously seen to have robust effects in CyclinA2- cells without a specific requirement for proliferation, these experiments were conducted on freshly thawed AML samples without growth factor pre-incubation. As expected at both 2 h and 6 h post-thaw, AML samples displayed low percentages of CyclinA2+ cells (2 h average: 3.41%, 6 h average: 3.8%) and good post-thaw viability with live, non-apoptotic leukemic cell frequencies averaging 57.2% and 58.0% at 2 h and 6 h in unstimulated conditions. Treatment with etoposide for 2 h or 6 h had no effect on the percentages of non-apoptotic cells (data not shown). Induction of each DDR readout was examined in response to etoposide treatment to assess the integrity of signaling along the DDR pathways, and the inter-relationships between signaling pathways. Flow plots illustrating the gating scheme used and examples of etoposide responses in AML samples are shown in Figure 9. At both 2 h and 6 h co-activation of repair proteins within unique DDR pathways was observed following etoposide treatment (Figure 9). Stronger correlations were observed for readouts within a single pathway (HRR pathway: p-ATM vs. p-BRCA1) than for readouts from distinct pathways (HRR pathway: p-BRCA1 vs. NHEJ pathway: p-DNA-PKcs). Comparison of NHEJ (p-DNA-PK) with HRR (p-BRCA1) readouts in individual samples (top right panel) identified AML samples with lower activation of both repair pathways, samples with robust activation of both pathways, and others which predominately activate NHEJ, suggesting that individual leukemia samples vary in the magnitude of activation of specific DSB repair pathways (Figure 9).

## Discussion

Genomic instability is a hallmark of cancer. Quantitative functional analysis of genomic instability could significantly improve risk assessment for cancer development in healthy individuals and characterize the DNA repair capacity of established cancers as a method for determining individualized therapeutic strategy. In order to achieve this, a more comprehensive, functional understanding of DNA repair pathways and the ability to quantify these pathways in samples from individual patients is needed. Moreover, functionally profiling DNA damage and repair pathways induced or targeted by specific agents will enable mechanistic characterization and differentiation of distinct drug classes [20,21].

The ultimate capacity of cells to respond to DNA damage-inducing therapeutics is potentially affected by the accumulation of multiple individual mutations in distinct components comprising DNA damage repair pathways. Functional assays, such as SCNP, can uniquely enable quantification of the physiological consequences of these alterations, which converge at the level of pathway and network responses. As such, SCNP can simultaneously highlight both the mechanisms of action for specific drugs as well as characterize the DNA repair capacity of samples from individual patients. This approach may complement sequencing-based mutational analysis of DNA repair genes, such as *BRCA1/2* sequencing, where many mutations have unknown function, epigenetic and/or non-coding alterations may not be detected, and the combined effects of pathway mutations are not known or understood [22].

By applying SCNP to simultaneously measure DNA damage and activation of multiple DDR pathways in distinct cell cycle subsets, and by using cell lines with known mutations in DDR signaling, data from the current study support the following major conclusions: First, while the ability to measure p-H2AX, p-ATM, p-Chk2 and p-DNA-PKcs using flow cytometry is well established [7,23-28], these data provide examples of methods to measure activation of additional DNA repair proteins from repair pathways including NHEJ (p-53BP1) and HRR (p-RPA2, p-BRCA1) using flow cytometry. Second, by controlling for proliferation rate through a focused SCNP analysis of CyclinA2+ cells, these data demonstrate the ability to functionally identify and differentiate cells with partially impaired (*BRCA1*+/-) or completely defective (*BRCA2*-/-) HRR repair machinery. For future studies, It is possible that this ability to control for proliferative state could be further improved by the use of additional markers beyond CyclinA2 such as CyclinE to mark G_1_ cells or p-Histone H3 to mark cells in M phase cells. Third, these data demonstrate that primary AML samples quantifiably differ in their relative activation of HRR vs. NHEJ components. This ability to differentiate unique AML signatures based on DDR pathway activation may have therapeutic implications; additional studies using clinically annotated samples to enable correlation with patient clinical responses to specific agents are planned.

The experiments described in this analysis were performed on cryopreserved AML samples. High concordances between both cell health and functional responses comparing fresh vs. cryopreserved AML samples have previously been established [29], supporting the potential utility of the current assay for the analysis of freshly isolated samples. Studies to confirm this are planned.

These findings are consistent with pre-clinical data showing that PARP inhibition for the treatment of cancers is most effective in *BRCA*-deficient or mutant samples. The absence of a functional HRR pathway forces the cell to use the error-prone NHEJ repair of DNA damage, which ultimately results in the accumulation of misrepaired DNA, complex DNA rearrangements and cell death [15,30-33]. Importantly, PARP inhibitor sensitivity has also been associated with lower expression of genes for additional DNA damage repair proteins beyond *BRCA1*[34], further supporting the need to holistically examine the functionality of the entire DNA repair network in order to accurately identify cellular competence for repair of DNA damage.

Another recently described method for assessing HRR function in single cells, which has generated useful data in cell lines, measures repair and reactivation of an HRR substrate reporter gene (DR-GFP) [35]. However, the requirement for transient transfection in this system challenges the potential clinical utility of this approach [3]. Assays measuring RAD51 foci by microscopy have also been described as a measure of HRR function and our data suggest that these assays could be improved by specifically analyzing RAD51 foci in CyclinA2+ cells. SCNP, in contrast to these techniques, enables the simultaneous analysis in primary cells of multiple DDR signaling nodes in multiple cell subsets, providing a signature of the functional consequences of genetic mutations, without the need for any cell manipulation or subset isolation.

These data illustrate the capability to quantifiably measure functional activation of the 2 major DNA DSB repair pathways, NHEJ and HRR, both of which are relevant to genotoxin responses and cancer predisposition. As mutations in additional DNA repair pathways [such as nucleotide excision repair (NER), base excision repair (BER), and mismatch repair (MMR)] are also associated with cancer predisposition and/or drug sensitivity [3], the future development of functional assays for these additional repair pathways is also clinically relevant. The cell line data presented here establish that DNA repair deficiencies, including HRR haploinsufficiency, are detectable and quantifiable through functional assays, a notion recently supported by independent data [36]. Experiments using PBMC samples from healthy donors or patients with known germline mutations in the HRR pathway are needed to validate the clinical utility of these functional assays. Such analyses could form the basis for the development of screening tests to identify subjects at higher risk of developing cancer or stratification tests to inform on cancer patient selection for treatment with specific agents, such as PARP inhibitors.

This study demonstrates the utility of SCNP to functionally quantify multiple DNA damage-associated readouts in single cells of distinct cell cycle subsets and identify samples with defective DNA DSB repair capacity, including inherited haploinsufficiency. As multivariate analyses of SCNP data have proven useful in the prediction of clinical outcome in other oncology settings [37,38], future experiments with larger numbers of clinical samples are warranted in order to capitalize on the multidimensional nature of these data.

## Competing interests

DBR, LYL, BL, JAC, AC, REH, and AC are employees of and/or stockholders in Nodality, Inc.

## Authors’ contributions

DBR designed, performed, and analyzed the experiments and prepared the manuscript. LYL, JAC and AC performed experiments and analyzed data. BL analyzed and interpreted the data. REH and AC designed the study, interpreted data and prepared the manuscript. IS and SZF helped interpret the data and prepare the manuscript. All authors read and approved the final manuscript.
